# Partial Migration and Transient Coexistence of Migrants and Residents in Animal Populations

**DOI:** 10.1371/journal.pone.0094750

**Published:** 2014-04-10

**Authors:** Navinder J. Singh, Kjell Leonardsson

**Affiliations:** Department of Wildlife, Fish and Environmental Studies, Swedish University of Agricultural Sciences, Umeå, Sweden; Institut Pluridisciplinaire Hubert Curien, France

## Abstract

Partial migration, whereby a proportion of the population migrates, is common across the animal kingdom. Much of the focus in the literature has been on trying to explain the underlying mechanisms for the coexistence of migrants and residents. In addition, there has been an increasing number of reports on the prevalence and frequency of partially migratory populations. One possible explanation for the occurrence of partial migration, which has received no attention in the literature, is that of ‘transient coexistence’ during the invasion phase of a superior behaviour. In this study we develop a theoretical basis for explaining partial migration as a transient coexistence and derive a method to predict the frequency of residents and migrants in partially migrating populations. This method is useful to predict the frequencies of migrants and residents in a small set of populations as a complementing hypothesis to ‘an Evolutionary Stable Strategy (ESS)’. We use the logistic growth equation to derive a formula for predicting the frequencies of residents and migrants. We also use simulations and empirical data from white perch (*Morone americana*), moose (*Alces alces*) and red deer (*Cervus elaphus*) to demonstrate our approach. We show that the probability of detecting partial migration due to transient coexistence depends upon a minimum number of tracked or marked individuals for a given number of populations. Our approach provides a starting point in searching for explanations to the observed frequencies, by contrasting the observed pattern with both the predicted transient and the uniform random pattern. Aggregating such information on observed patterns (proportions of migrants and residents) may eventually lead to the development of a quantitative theory for the equilibrium (ESS) populations as well.

## Introduction

Animal migration is a widely known phenomenon where individuals move between seasonal ranges on an annual basis [1]. Animals migrate seasonally to favourable habitats to overcome the severity of climate, escape predation, competition or disease, or for better foraging opportunities [2], [3]. These migrating individuals transport nutrients, genes and biomass, causing ecosystem level impacts [4], [5]. Research on migration is therefore of vital importance. A key observation in the past five decades that has emerged from the migration literature is that many migratory populations are only partially migratory, i.e. only a fraction of the population migrates [6]–[9].

A number of explanations have been suggested for the causes of partial migration and its maintenance in nature [10], [11]. Chapman et al. [12] reviewed the existing literature on the ecology and evolution of partial migration and reported general mechanisms to explain its occurrence. First, existence of residents and migrants may be mediated by density dependence in the non-shared habitats implying a total density below the carrying capacity in the shared habitat [13]; secondly, migratory and non-migratory behaviours may be genetically controlled through parental bet hedging, or a genetic dimorphism [6], [12], [14]. Finally a mixed evolutionary stable strategy (ESS) arising from a ‘frequency dependent selection’ may allow “coexistence” of the behaviours [7], [15], [16]. Several mechanisms may allow the equilibrium to be maintained and ‘conditional strategy’ dependent on the life history (age, sex, body mass), boldness, behaviour and frequency has received much attention lately [8], [10], [11], [17]–[20].

Besides the above-mentioned mechanisms, there is an additional and complementing explanation for the coexistence, is a ‘transient’ phenomenon. This has received little attention probably because it is assumed to be extremely infrequent. When a new behaviour (e.g. resident or migratory) enters a population, it will increase in frequency as long as it is superior to the existing behaviour. When the the frequency increase in a population as long as the fitness of individuals expressing one behaviour is higher than the fitness of individuals showing the other behaviour, one will eventually replace the existing one. Thus, there will be a time period when both behaviours can be observed and this type of transient coexistence could be mis-interpreted as partial migration at equilibrium if the transient phase is very long. This logic can also be followed in the way, that partial migration could be an early stage in the evolution of migration [3], [21]–[23].

The existing explanations focus on the mechanisms behind the existence of two behaviours maintained at an equilibrium [10], [11]. The assumption of equilibrium doesn't easily allow predictions of the frequencies of the two behaviours in different populations since their equilibria and densities may differ for example due to environmental heterogeneity. In contrast, an explicit frequency distribution can be derived for transient coexistence, when the invasion of a new behaviour follows logistic growth and when the selection for it, is density independent. This invasion may arise due to any of the existing mechanisms reviewed in Chapman et al. [12], but for a single population, the new equilibrium ends up at either of the extreme ends, at a frequency of zero or one.

We demonstrate how an assumption of transient coexistence of partial migration can be used to derive a probability density function and the associated cumulative distribution function to predict the expected frequency of migrants and residents in naturally occurring populations. We also apply this approach to empirical data on one aquatic and two terrestrial species.

## Model formulation

We use the logistic growth equation to derive an expected frequency distribution of the two behaviours. We restrict the analyses to cases where there is density dependence in the shared habitat (breeding or non-breeding). We further assume that the population growth rate follows the classical logistic growth curve, and that the total population is at or close to its carrying capacity. The analytic derivation of the frequency distribution is simplified by the fact that the logistic function is symmetric in its S-shape. We show that small to moderate deviations from this assumption will lead to negligible changes in the predicted frequency distribution.

**Figure 1:**
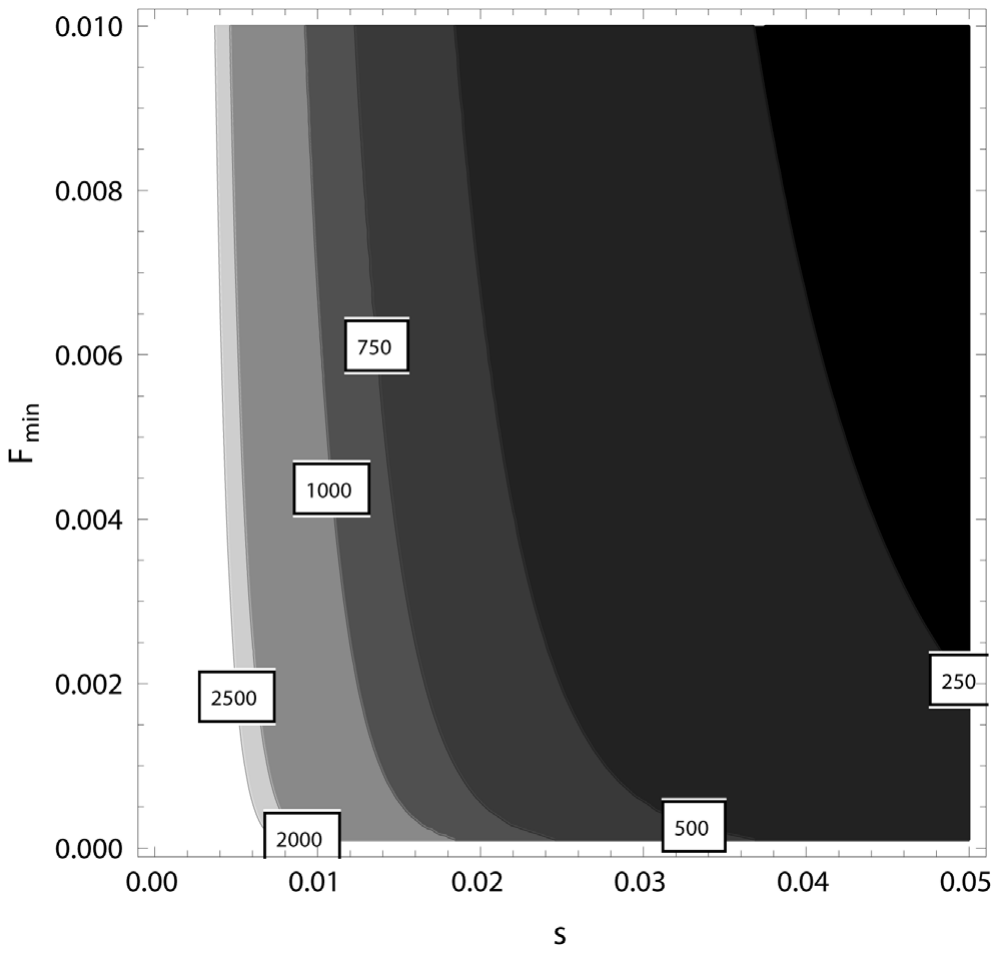
The expected transient time in years for a superior invader to increase in the original population from the fraction *F*_min_ to the fraction 1−*F*_min_, where *s* is the rate of spread (fitness) of the superior invader and *F*_min_ is the smallest frequency at which one can observe the behaviour (user defined).

Furthermore, we restrict the observations between defined endpoints of the frequency spectrum; from *F*_min_ to 1−*F*_min_, where *F*_min_ is the minimum frequency at which we can observe the least common behaviour (either migratory or resident). The frequency (*F*) of the least common behaviour will be Min(*F*, 1−*F*) ≥*F*_min_. It does not matter if the least common behaviour (resident or migratory) is superior or not, which simplifies the observation process. We start the derivation with the classic logistic growth function:
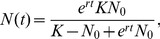
(1)Where *r* describes the intrinsic rate of natural increase, *K* is the carrying capacity, and *N*_0_ is the (invaders') initial population density, to accommodate the invasion of the alternate behaviour in a population initially consisting of either residents or migrants. Even if the population growth is density dependent, the selection for a superior behaviour is considered density independent [24]. Under density independent selection, the spread of the invader in the population will occur at a rate *s*, which is the difference between the *r-values* for each of the behaviours. Therefore, to avoid confusion in terminology we replace *r* with *s*. Next we divide both the left and the right hand side of equation 1 by *K* to make the equation dimensionless. Dividing both the numerator and the denominator on the right hand side by *K*, and replacing *N*_0_/*K* with *F_min_*, leaves us with an equation for 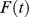 (equation 2). The function 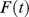 describes the fraction of the population with the invading behaviour at time *t*. (see Eq. 2).
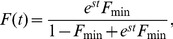
(2)Since the rate of spread, *s*, is likely to be much smaller than the intrinsic growth rate, we expect the transient times from *F*(t) = *F*_min_ to *F*(t) = 1−*F*_min_ to exceed the generation time by several orders of magnitude, which can be seen in Figure 1. For this graph the transient time, *t*, was solved from equation 2 by setting *F*(t) = 1−*F*_min_. Thus, partial migration may exist as a transient coexistence for long periods of time (about 200 to 2000 years in this hypothetical case, Figure 1) and could be mistaken for other explanations. This is especially important to consider when exploring the causes that determine the proportion of migrants and residents in populations.

### Frequency of migrants and residents in multiple populations

Often, our interest lies in determining if a species is partially migratory [12]. To determine that requires measuring partial migration in multiple populations of that species. Therefore, in order to derive the expected frequency distribution of the proportion of the least common behaviour in multiple populations, the fraction of the least common behaviour needs to be considered as a random variable (stochastic process with random variation). For any population with partial migration, the frequency of the least common behaviour needs to be associated with a probability of occurrence and that the frequencies are independent among populations. This criterion is fulfilled if we assume that the mutations or the behavioural changes arise independently over time in different populations. However, it should work also in situations when a mutant arises in one population and then spreads to other populations over time. As a consequence, the invasion growth curves will originate and occur independently between populations over time. Hence, collecting data to calculate *F*(t) for different populations will eventually give enough data to produce a histogram of the expected frequencies of the least common behaviour. In order to make *F*(t) comparable between populations we need to remove the dependence on *s*, since we do not necessarily expect *s* to be the same in different populations. Moreover, it would be difficult to estimate the magnitude of *s*. To cancel out *s*, we create a new variable that fulfills the properties of a cumulative distribution function (CDF). The CDF accumulates the probabilities from zero (at *F* = *F*_min_) to 1 (at *F* = 1/2). The upper limit at *F* = 1/2 condition is fulfilled because the least common behaviour can by definition never exceed half the population. To derive the CDF we replace *F*(*t*) by *F*(*X*) and solve for *X* to have *X*(*F*). Then we let *X*(1/2) = 1, which give us CDF(*F*) = *X*(*F*)/*X*(1/2).

**Figure 2:**
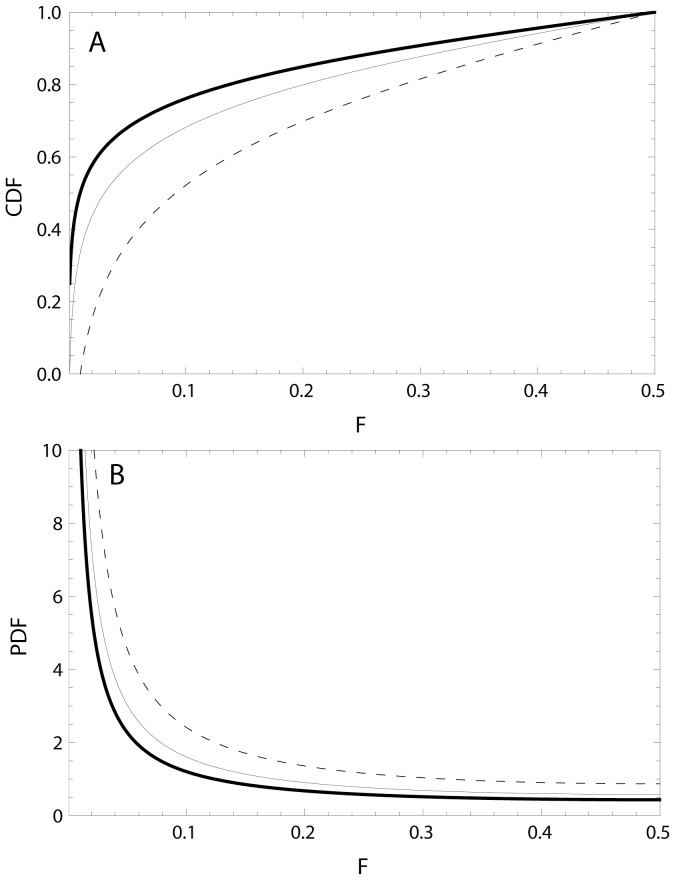
Expected cumulative distribution function (CDF) (A) and the probability density function (PDF) (B) of the less common behaviour among populations with partial migration (frequencies between *F*_min_ and 1−*F*_min_ of the less common behaviour) as a function of the observed frequency of the less common behaviour (F). The thick line denotes *F*_min_ = 0.0001, thin solid line denotes *F*_min_ = 0.001, and the dashed line denotes *F*_min_ = 0.01.

The CDF is defined in the range *F*_min_≤*F*≤1/2, and the mathematical function for the final CDF is independent of *s* (equation 3).
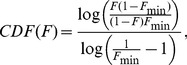
(3)The probability density function (PDF) is the derivative of the CDF with respect to *F*, which becomes (equation 4):
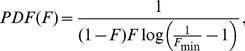
(4)Either of these functions can be used to fit to empirical data, but the PDF is preferable in such situations since it can be used to calculate the loglikelihood values from the data. The deviations from the patterns produced from equation 4 (Figure 2) should indicate the presence of partial migration explained by causes other than transient dynamics of superior invaders. It is important that *F*_min_ is the same for all populations included in a single empirical study, if not equation 3 and 4 will no longer be valid. Moreover, when the least common behavior occurs at very low frequencies, partial migration may be difficult to detect in some populations, meaning that F_min_ should not be set too low. In the application to real data we show how *F*_min_ values can be selected.

### Sensitivity analysis of the symmetric growth assumption

A requirement for the invading behaviour to increase in a population is that the fitness in terms of carrying capacity for this behaviour is higher than that of the existing one. With increasing difference between the two *K*'s, the initial increase of the invader will be slower than the final disappearance of the original behaviour. The classic logistic function will therefore give biased results when *K*'s are very different; however, is this bias negligible for reasonable differences in *K*-values? To estimate the magnitude of bias in our predictions, we tested three ratios of *c* = *K*_Invader_/*K*_Original,_ in the differential equations describing population growth (equations 5), where both behaviours followed logistic growth, and ran Monte Carlo (MC) simulations.
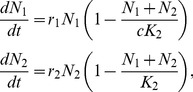
(5)The parameters *r* and *K* were allocated uniform random numbers for each run, and the ratio *c* was set to 1.005, 1.5 and 5.0 respectively in each of three MC simulations, each based on 500 000 runs. The *r*-values ranged from 0.05 to 1.5 and the *K*-values ranged from 1000 to 100000. The initial densities were set to N_Invader_ = F_Min_*K_Original_/100, and N_Original_ =  K_Original_-F_Min_* K_Original_/100, when integrating the differential equations. All simulations were run until the proportion of invaders (*P*_Invader_) exceeded 1- *F*_Min_, and the result for each run was extracted at a single random time that fulfilled the criterion; *F*_Min_ ≤*P*_Invader_≤1−*F*_Min_. Frequency distributions of *F* were compiled for each of the three *c*-values, for comparison with the frequency distribution predicted using the classic logistic equation (Equations 5).

**Figure 3:**
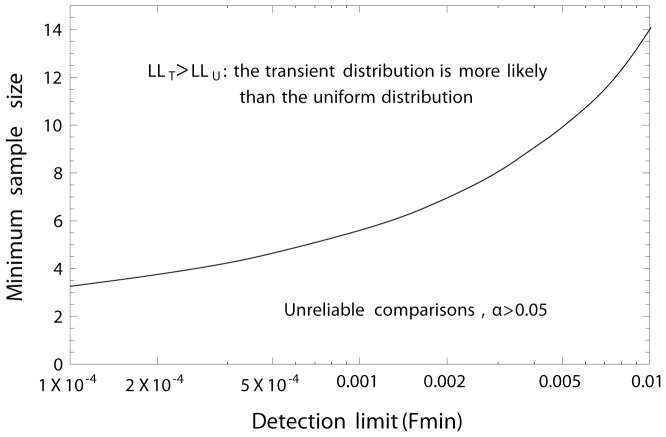
The transient distribution describes the data better than the uniform distribution when the log-likelihood of the data based on the transient distribution (LLT) exceeds the log-likelihood based on the uniform distribution LLU and α<0.05.

The analysis of the potential bias in the predictions when comparing with simulated results based on differences in K-values between the invader and the original behaviour showed that the bias was negligible in the range of *K*-ratios that could be of relevance (Figure 3). There was almost no noticeable bias for ratios between the carrying capacities in the range 1.005≤c≤1.5 when inspecting the simulated and the predicted frequency distributions (Figure 3). At c = 5 the bias is observable, showing slight over-estimated predictions at the lowest F-values and a small over-estimation at higher F-values. In terms of differences in the relative frequencies, these over-estimations were less than 0.001 for F-values above 0.1 when using a class width of 0.01.

## Applications

**Figure 4:**
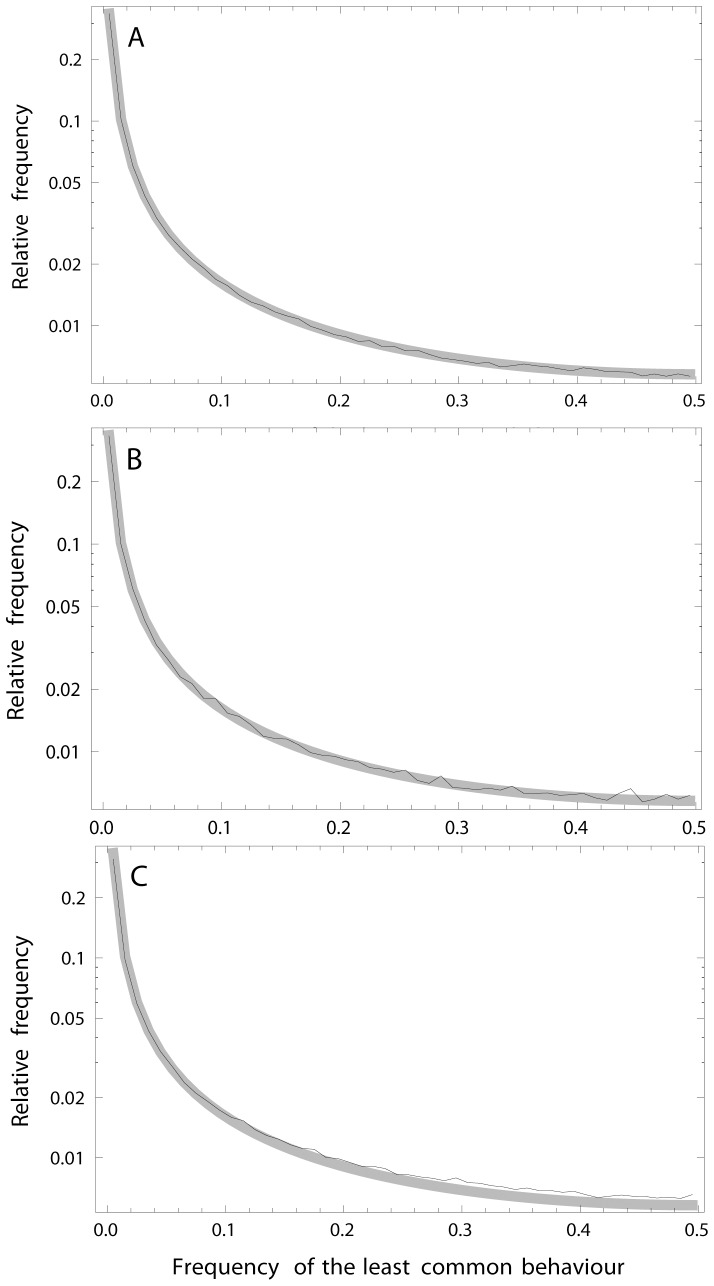
Comparison between the predicted frequency distribution of the least common behaviour (*F*), center of the thick gray line, and distributions obtained from Monte Carlo simulations (thin black lines) where the ratio, *c*, between the invaders' and the original behaviour' carrying capacities varied from A) *c* = 1.005 solid line, B) *c* = 1.5 thin dashed line and C) *c* = 5.0 dotted line. Increasing values of *c* implies increasing asymmetry in the logistic growth curve. The large width of the predicted gray line is more for help with the visual interpretation.

To test for occurrence of transient dynamics, empirical data on the frequencies (*F*) of the “least common behaviour” can be used to compare the fit to each of the two distributions, the transient versus the uniform random distribution. In the absence of an existing explicit framework, we use the uniform random distribution as an alternative, i.e. as a null hypothesis, to represent the distribution of frequencies of the two behaviours from different populations. This comparison can be made by calculating the log-likelihood (*LL*) for the data based on each of the distributions probability density functions (PDFs). The distribution resulting in the largest *LL* has a better fit to the data since both distributions have exactly the same parameters, *F*_min_ and 0.5. The log-likelihoods are calculated as:
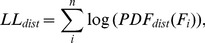
(6)where *F_i_* is the frequency of the least common behaviour in population *i*, and *PDF*_dist_ is the probability density function for distribution *dist*. The PDF for a uniform distribution in this case will be 1/(1/2−*F*_min_) for *F*_min_≤*F*≤1/2 and 0 elsewhere. It appears that when applying uniform random data in a Monte-Carlo simulation to compare the *LL* from the two distributions, reliable tests can be made with as few observations as 4, given that *F*_min_ is very low, ca. 0.0001. The reason why few samples are required to separate between the two PDFs at a low detection limit is that the PDF for the transient hypothesis is much steeper at low than at high F-values. With higher *F*_min_-limits more samples will be required for reliable separation of the distributions (see Figure 4).

The transient PDF describes the data better than the uniform distribution when the log-likelihood of the data based on the transient distribution (*LL*_T_) exceeds the log-likelihood of the uniform distribution (*LL*_U_) and α<0.05. When the reverse is true the uniform random distribution might still not be the most appropriate distribution. It rather means that the uniform random distribution describes the data better than the transient distribution. A standard distribution test like a Kolmogorov-Smirnov test can be used to test if the data was drawn from a uniform distribution (H_0_) or not (H_A_). If H_0_ is rejected then there may be aggregations of frequencies at some *F*-values, but so far there is no existing theory to explain the existence of aggregations at some intermediate *F*-values.

### Application to real world data

The framework we develop above either needs data on proportion of residents and migrants from more than a single population, or from the same population over time. We apply the framework on multiple populations/subpopulations assuming that the new behavior likely arose from a single brood from which the offspring might have dispersed and established at a new site away from the birthplace. Over time the population will therefore have several hotspots with different proportions of migrants and residents depending upon the time of arrival of the “invading” behaviour. In such situations, data on partial migration from different subpopulations should fit our predictions when the partial migration is a result of a transient coexistence rather than at equilibrium. Here we use three examples based on real world data on partial migration to show how predictions on transient coexistence can be made using our framework. We compiled population level estimates of the proportion of migrants and residents from three species: white perch (*Morone Americana*, N = 6 populations, [25]), moose (*Alces alces*, N = 7, [26]), and red deer (*Cervus elaphus*, N = 6, [13]). These species were selected because multiple population level estimates of the proportion of residents and migrants were directly available. We selected the proportion of the least common of the two behaviours to explore the fit of the observed data against our predictions of transient coexistence. We calculated *F*_min_ as the harmonic mean of the ratios 1/*n* from all populations or sub-populations in each study respectively, where *n* denotes number of tagged or selected individuals in each population. This means that if 20 individuals are tagged in each population, then the least frequency that can be detected is 1/20. We calculated the Loglikelihood values using *equation 6*.

**Figure 5:**
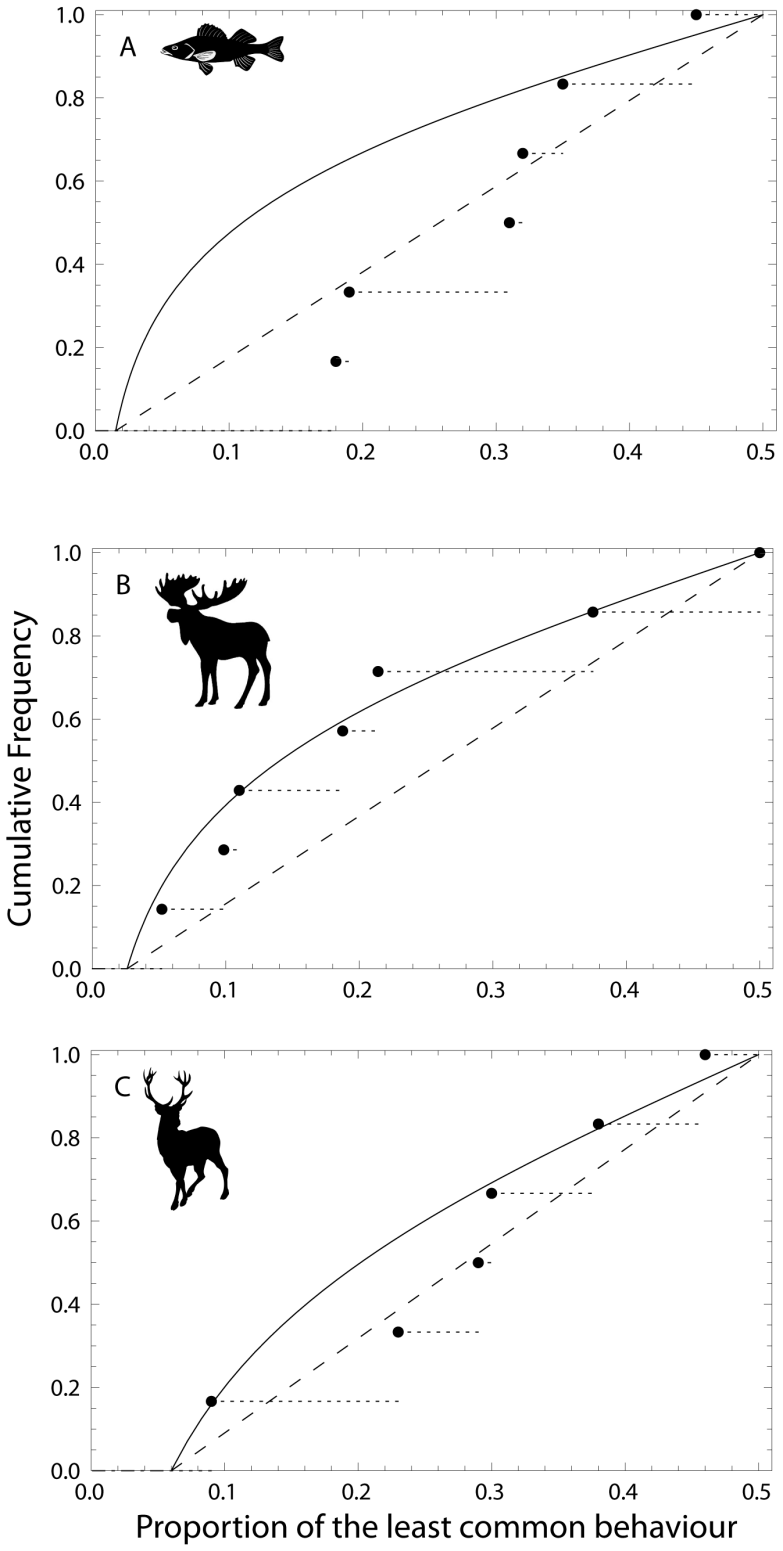
Cumulative distribution functions for the transient hypothesis for A) White perch (*Morone americana*) data from Kerr and Secor (2012) using F_min_ = 0.0151, B) Moose (*Alces alces*) data from Singh et al. (2012) using F_min_ = 0.026, and C) Red deer (*Cervus elaphus*) data from Mysterud et al. (2011) using F_min_ = 0.060. The dashed diagonal line denotes the cdf for the uniform distribution between F_min_ and 0.5.

For white perch, the Loglikelihood estimate was 1.14 for the transient PDF function based on *F*_min_ = 0.015, as opposed to 4.34 for the uniform distribution ∼U(0.015–0.5), n = 6 (Figure 5a). For moose, *F*_min_ was 0.026 and the estimate for transient PDF function was 5.25 in contrast to 5.23 for the uniform random distribution ∼U(0.026–0.5), n = 7 (Figure 5b). Finally, for red deer (n = 6 and *F*_min_ = 0.06) the estimates were 4.14 and 4.93 for transient PDF and uniform random distribution ∼U(0.06–0.5) respectively (Figure 5c). The transient case was supported in moose and not in either red deer or white perch.

## Discussion

Considering “transient coexistence” as an additional complementing hypothesis for the existence of partial migration allowed us to analytically derive a frequency distribution for the two behaviours, residency and migrants, among populations. This does not mean that the aim of this study was to explain that all partially migrating populations are in a transient state. The idea is rather to provide a framework in which the observed patterns of partial migration can be assessed, for example as another null hypothesis. We also show how the expected frequency distribution can be used to assess the transient versus a null hypothesis consisting of a uniform random distribution that will represent the equilibrium conditions in real populations until a quantitative theory is derived for these conditions.

In terms of log likelihood values, moose data was found to support the transient hypothesis whereas red deer and white perch did not. From a frequentist's perspective, none of the populations fulfilled the requirements for statistical inference due to either too low number of populations studied for each species, or alternatively that too few individuals were tagged or analyzed in each population. For the white perch analysis 17 populations would have been needed to allow a reliable comparison at α = 0.05. In contrast, staying with six populations would have required that 784 individuals from each population had been analyzed in the first place. For moose 26 populations would have been needed, or that 38 individuals had been tagged in each population. For the red deer the corresponding numbers should have been 60 populations, but increasing the number of tagged red deer would not have made a difference as long as the frequencies had remained the same. At least some of these numbers are not unrealistic, such as tagging 38 moose per population. Nevertheless, there are other methods such as repeated censuses that could be used to estimate the proportion of migrants and residents as these methods generate more observations than tagging studies. The main reason for having the proportions based on more observations is that it reduces the detection limit, which in turn reduces the number of populations needed for statistical inference. Otherwise, model selection approaches such as AIC/BIC could also be employed [27]. When applying a model selection approach the transient PDF fits the moose data better than did the PDF of the uniform random distribution. This comparison is straightforward since both PDFs have the same parameters, *F*_min_ and 0.5.

The existing explanation for the observed patterns of partial migration pattern in moose is that partial migration is an adaptation to the latitudinal variation in the climate [26]. However, there is also support for the transient coexistence hypothesis in moose due to the recent history of moose population and distribution changes, in Sweden and the Scandinavian region. Moose were almost extinct from a major part of the Scandinavian region during 1900s due to overhunting [28], [29]. Since then, the conservation and management actions for moose have resulted in its successful recovery from near extinction to its sustainable management. A number of recovery oriented measures were adopted during this period which involved extensive plantation of forage species, thereby creating favourable habitats, shortening of the hunting season, banning of passive and too effective hunting methods (such as pitfall traps, snares), reducing poaching, extirpation of large carnivores, age and sex specific harvest and adaptive management [30]. This 100-year period of change resulted in an impressive recovery of moose populations in Sweden from maybe a few hundred to around 200 000–300 000 moose today (after the hunting season). While the population recovered, the landscape underwent dramatic changes in terms of an increase in human population as well as commercial forestry practices and infrastructure development. This recovery of population also drove changes in distributions, requiring animals to adapt to local environmental conditions as they dispersed. This was shown in a recent study on partial migration of moose, which was also a source for the data for our paper [26]. Although red deer in Norway experienced a similar history, we did not find evidence for the support of transient hypothesis there [31]. This could probably be due to the data deficiency discussed above, or red deer have adapted well to the local environment and local factors maintain partial migration in populations at a dynamic equilibrium driven by food availability [13]. In the case of white perch, there was insufficient information about the history of the populations to make any inferences towards or against transient hypothesis [25]. Partial migration in perch was explained by local environmental differences and the life history of individuals [25]. Nevertheless, data deficiency as a limitation also emerges out from this study. Finally, from cases such as that of moose, it might be possible in future to explore the mechanisms behind evolution of migration, especially when relating the observations of partial migration to historical changes in selection pressures such as environmental conditions or human interventions [21], [23], [32].

### Method related aspects

There are method related aspects in the study that require explanation and justification. In order to cancel out *s* (the rate of spread) in the derivation, we assumed that the carrying capacity does not change when the superior behaviour invades. According to the simulations of an invasion of a superior behaviour, the predictions should still be applicable for empirical data when the carrying capacities for the two behaviours differ. Minor bias might be expected if the *K*_Invader_/*K*_Original_-ratios are very high, *c*≥5, but such magnitudes should be rare in nature. There are at least two reasons for this: first, it is unlikely that a recent invader can increase the carrying capacity significantly in the shared habitat. Secondly, if the ratio is large, then the invasion time will be relatively short and partial migration might not be observed.

The PDF and CDF that we derived were based on the assumption that all included populations were in a transient state. Therefore, a mixture of transient and equilibrium populations will create an intermediate fit. It is hence more likely, that a transient state may be observed within the same species, from only a few sub-populations, since the mutants are likely to spread to other populations in time.

For the method to predict the frequency of migrants and residents, it is important that a common *F*_min_ is set for all populations in the study. If *F*_min_ is set very low then the occurrence of partial migration needs to be detectable in any population at that level. In studies where individuals are tagged, the *F*_min_ equals 1/the number tagged, as long as the number of tagged individuals are the same in each population. A solution for the case, when the number of tagged individuals is not equal across populations, could be to use the harmonic mean of the 1/*n* ratios, as we did in the three presented examples. Alternatively, if the smallest 1/n ratio is less than the harmonic mean, then it should be used instead, since empirical ratios below *F*_min_ will produce erroneous results.

Another contribution of our framework is that it might provide information on how common or uncommon “mutant invasions” are in natural populations [33]. A common assumption is that these events are extremely rare [34] (but see Pulido & Berthold [33]). However, once they arise they may persist over a long period of time. Based on this assumption, a human lifetime is much shorter compared to the expected transient times due to low fitness differences between the traits (Figure 1). These long periods of invasion could be misinterpreted for other explanations of coexistence of migrants and residents [7], [12]. Thus, if the empirical data speaks in favor of transient coexistence in more than a few species, then it should be a reason to reconsider the rate of “mutant invasions”, especially in a rapidly changing environment [33], [35]. However, if it turns out that the transient hypothesis is frequently supported, then a more probable explanation should be that the behaviours for some reason are balanced according to the equilibrium theories at low frequencies of the least common behaviour, as a rule rather than an exception. A particularly interesting question is, if there are general patterns in frequencies of these behaviours among populations and across species. If so, it should be possible to find some general explanation about the mechanisms for this dominating pattern. In this sense our study provides an initial platform that can be further developed as empirical evidence is gathered that speaks in favour or not of the transient hypothesis.

## Acknowledgments

Luis-Miguel Chevin and three anonymous reviewers provided valuable comments, which greatly improved the manuscript. Göran Spong and Andrew Allen gave valuable advice on writing.
